# Epidemiological and clinicopathological profile of pancytopenia: A cross-sectional study

**DOI:** 10.6026/973206300223348

**Published:** 2026-06-30

**Authors:** Chethan N, Divyansh Badole, Zeeshan Farooqui, Manoj Gupta, Sachin Parmar

**Affiliations:** 1 Department of General Medicine, MGMMC & MYH Hospital, Indore, Madhya Pradesh, India; 2 Department of Community Medicine V.K.S. Govertment Medical College, Neemuch, Madhya Pradesh, India

**Keywords:** Pancytopenia, vitamin B12 deficiency, clinico-haematological profile

## Abstract

Broad etiology of pancytopenia also makes it regionally heterogeneous, requiring a multifaceted clinico-haematological assessment. ence, this cross-sectional study at MGM medical college and Maharaja Yeshwantrao hospital in Indore involved 200 patients whose CBC, peripheral smear, LFT/RFT, vitamin B12/folate levels and bone marrow were measured within a period of 12 months and had to be above 15 years old with pancytopenia (not under chemotherapy/radiotherapy). Severe anaemia (53.5%), moderate leucopenia (34%), severe thrombocytopenia (25.5%) and 31-40 year-olds had the highest (21.5%) were severe cases; pallor (60%) and dyspnoea (32.5%) were most common. Top causes were vitamin B12 deficiency (28.5%), hypersplenism (12%) and dimorphic anaemia (10.5%) and gender-B12 association had a significant value (p=0.028). Thus, the major reversible cause is nutrition deficiencies, especially the vitamin B12 deficiency, which should be screened and supplemented by communities.

## Background:

Pancytopenia, a diverse disorder, lowers erythrocytes, leucocytes and platelets to abnormal levels. It can indicate benign, systemic and malignant diseases [1]. Haemoglobin under 13 g/dL in males and 12 g/dL in females, total leucocyte count under 4000 / cu mm and platelet count under 150,000 / cu mm, which vary by age, sex and clinical setting [2]. The dominant mechanism controls bone marrow morphology and diagnostic/therapeutic interventions [3]. Tertiary care centres in sub-Saharan Africa, South Asia and the Middle East report very different aetiological profiles, highlighting the importance of region-specific epidemiological data [4]. A prospective, cross-sectional, cohort study in India of 139 consecutive cases of nutritional megaloblastic anemia found that 80.5% had concomitant thrombocytopenia and 43.8% had neutropenia and thrombocytopenia, proving that nutritional deficiency can cause pancytopenia [5]. Pancytopenia in North Indian hospital-based patients is mostly caused by vitamin B12 deficiency and vegetarian diets and poverty [6].Aplastic anaemia, with hypocellular marrow and peripheral pancytopenia, is the main cause in 10% to 52% of pancytopenic patients, depending on study population and referral pattern [7]. Aspiration cytology and trephine biopsy can distinguish aplastic, megaloblastic and infiltrative and dysplastic bone marrow pathologies in up to 77% of pancytopenic cases [8]. Some diseases like HIV, tuberculosis, kala-azar, falciparum malaria and sepsis can be treated to reverse tropical pancytopenia. Chronic liver and kidney disease cause hypersplenism, cytokine production impairment and erythropoietin production, causing multilineage cytopenias [9, 10]. These studies are increasing, but institution-specific research is needed to maximize locally relevant diagnostic algorithms because pancytopenia's aetiology, clinical presentation and haematological parameters vary by region, hospital type and referral pattern [11, 12]. Therefore, it is of interest to study epidemiological and clinico-pathological characterisation of pancytopenia at a tertiary care should be carried out in a comprehensive manner to come up with evidence-based clinical practice focused on the local disease burden.

## Materials and Methods:

This observational and cross sectional study was undertaken in the department of medicine in Mahatma Gandhi Memorial (MGM) Medical College and Maharaja Yeshwantrao (M.Y.) Hospital, Indore, the central region of India where all treatments and research services are given to the patients at no cost. The research was conducted in the course of 12 months, between March 1, 2023 and February 29, 2024. Two hundred patients meeting the diagnostic criteria of pancytopenia, which include haemoglobin of less than 13.5 g/dL in males and less than 11.5 g/dL in females, total leucocyte count of less than 4000 cells/mm3 and platelet count of less than 150000/mm3 and aged over 15 years were recruited at the medicine wards, the Intensive Care Unit (ICU) and the outpatient departments (OPD) of M.Y. Hospital and its affiliates. The study excluded patients who did or were undergoing cancer chemotherapy/radiotherapy. All the enrolled patients were carefully assessed based on systematic assessment that included detailed demographic profiling (age and sex), detailed medical history including onset and duration of complaints, history of chronic medical or surgical disorders, relationship with previous and current drugs, family history and personal history including dietary, sleep and bowel or bladder habits. The through clinical observation was conducted (general physical examination and systemic cardiovascular, respiratory, gastrointestinal and neurological system). Routine lab tests were a complete blood count (CBC), peripheral blood smear analysis, reticulocyte count, liver and kidney function tests (LFT and RFT). Invasive procedures like serum Vitamin B12, serum folate, bone marrow aspiration and biopsy were done on clinically indicated basis and informed written consent was taken before such invasive procedures. All the collected data were inputted and analysed by the help of proper statistical software, categorical variables in the form of frequencies and percentages, continuous variables summarised with the help of median and standard deviation (SD) and Chi-square test was performed to compare results with categorical variables with the help of a p-value of less than 0.05 as the statistically significant value. The protocol of the study was also reviewed and approved by the Institutional Ethics Committee before the start and the study was carried out as per the Declaration of Helsinki and the institutional guidelines and the written informed consent of all the participants or its legally permitted representatives was also taken and the confidentiality of patient information was preserved throughout the study.

## Results and Discussion:

The current study recruited 200 children with pancytopenia, of which 60 per cent were male and 40 per cent were female (Table 1). Similar to aforementioned studies, Fazal *et al.* [13] Gayathri and Rao [4] Khunger *et al.* [8] also documented a predominance of male patients in their respective series of patients with pancytopenia. In this study, the highest numbers of patients were productive working people. The maximum proportion was found to be in the age group of 31-40 years (21.5%) and 21-30 years (20.5%). Kiya *et al.* [9] similarly observed that young to middle-aged adults are the most common age group pancytopenic presentation in an Ethiopian tertiary care centre. They attributed this finding to increased exposure to infectious causes and nutritional deficiency. Significantly, 17% of patients in our cohort were above 60 years of age and harboured systemic comorbidities like CKD, CLD and haematological malignancies in contrast to mean age at diagnosis of 49.4 years in Vargas-Carretero *et al.* [12] which reflects higher burden of myelodysplastic syndrome in Mexican tertiary care. The most significant haematological abnormality was found to be severe anaemia (haemoglobin < 4.9 g/dL) in 107 (53.5%) patients (Table 2) which reflects the impact of nutritional deficiency and infiltration of marrow. Haemoglobin of 5-only 4.5% had 7.9 g/dL and 8-10.9 g/dL and none had 11 g/dL or more. Kiya *et al.* [9] recorded severe anaemia in 57% of patients diagnosed with pancytopenia. This observation by them further reaffirmed geographical consistency in findings. Moderate leucopenia was the most common leucocyte abnormality, with 34% of patients having a total leucocyte count of 2001-3000/cu mm and 32.5% of patients having a total leucocyte count between 3001-4000/cu mm. Moderate leucopenia was reported as the most common white cell finding in pancytopenic patients by Gajbhiye *et al.* [3] In the current series, severe leucopenia was rare as expected because profound neutropenia has been shown to be related to certain marrow failure states such as aplastic anaemia and acute leukaemia. The platelet counts showed a wide variability, with 25.5% patients having severe thrombocytopenia (< 20,000/cu mm), while 26.5% patients had platelet counts between 40,001-80,000/cu mm (Table 2). Cordan and Tukek [1] found a much lower rate of severe thrombocytopenia (6.3%) in their Turkish population, possibly due to a difference in referral pattern, nutritional megaloblastosis burden and distribution of underlying aetiologies. In another study, Gajbhiye *et al.* [3] showed that 42% of their patients had platelet counts of 25,000/mm³, which is more reflective of the thrombocytopenic burden seen in this study. The most common presenting complaing was generalized weakness in 120 patients (60%) which shows anaemia as universal cause of fatigue in virtually all major causes of pancytopenia (Table 3). Sixty-five patients (32.5%) had dyspnoea and pallor due to the haemodynamic effects of severe anaemia. Knuckle hyperpigmentation which is a typical skin condition associated with vitamin B12 deficiency was reported in 54 patients (27%). This is a useful diagnostic clue given the predominance of megaloblastic aetiology in these patients. Additional findings were giddiness (24%), splenomegaly (14%) and loss of appetite (19.5%), fever (11.5%) and bipedal oedema (9.5%). The latter is characteristically related to hypoproteinaemia in CLD and severe deficiency states. In their Western Indian series, Patel and Prajapati [10] similarly described a comparable clinical profile due to nutritional deficits. The most common aetiology in the present study was vitamin B12 deficiency among 57 patients (28.5%) (Table 4) which is present in a good number in the Indian subcontinent. Jain *et al.* discovered vitamin B12 deficiency in a whopping 58% of their North Indian vegetarian cohort, blaming this on caloric inadequacy. Megaloblastic and dimorphic anaemia together constituted the major nutritional burden. In this regard, Bhushan D *et al.* [14] reported a reverse order of etiological up gradation with aplastic anaemia being the most common 31.5% followed by vitamin B12 deficiency and infection 19.2% and 13.7% respectively. In the current study (12%), Hypersplenism was the second most common cause, followed by dimorphic anaemia (10.5%) and combined vitamin B12 and folate deficiency (10%). The nutritional deficiencies involving megaloblastic and dimorphic anaemia were also cited as dominant aetiologies by other regional Indian studies, including Patel and Prajapati [10] from Western India and Khunger *et al.* [8] in their pan-Indian series. This reinforces the nutritional burden underlying pancytopenia across the subcontinent. The frequency of HIV infection was seen in 16 cases (8%) followed by CKD 6.5%, sepsis 6%, aplastic anaemia 5.5%, CLD 5%, hypothyroidism 4.5%, and malaria and MDS each 2.5%. Gajbhiye *et al.* [3] reported cases of aplastic anaemia and CLD-associated pancytopenia, which strengthens the link between hepatic disease, portal hypertension, and hypersplenism. Hypothyroidism is a rare but well-known reversible effect of antiretroviral therapy (ART). Malaria and myelodysplastic syndrome (MDS) must be considered as differentials in tropical countries stated by Vargas-Carretero *et al.* [12]. The association observed between gender and Vitamin B12 (Cobalamin) deficiency was statistically significant (p =0.028) (Table 5). Male patients were more likely to be deficient than female patients, likely due to differences in diet, alcohol consumption and medications that impair cobalamin absorption. According to Jain *et al.* [6] there was no gender-based difference; however, the deficiency rate was high due to their high vegetarian diet which was not a function of gender. They did find this result which may be due to their population. Statistical association done using Chi-square test; Age group was significantly associated with generalized weakness (p=0.05). Younger and middle-aged adults are likely affected due to combined impact of malnutrition and infective burden. Pallor, on the other hand, did not show a statistically significant age association (p = 0.75). This is a clinical feature which apparently shows up in practically all categories and age groups of pancytopenic patients, as also reported uniformly by Gayathri and Rao [4] and Bhushan *et al.* [14].

## Conclusion:

Vitamin B12 deficiency accounts for 28.5% of pancytopenia cases, with a statistically significant male preponderance and highest burden in productive working-age groups. In this central Indian tertiary care population, micronutrient deficiency plays a major role in pancytopenia pathogenesis, as 49% of cases had nutritional aetiologies. Developing targeted, evidence-based diagnostic and therapeutic algorithms for local disease burdens requires institution-specific pancytopenia epidemiological profiling.

## Figures and Tables

**Table 1:** Demographic profile of the study participants

**Category**	**Variable**	**Number (n)**	**Percentage (%)**
Gender	Male	120	60
	Female	80	40
Age Group (Years)	15-20	30	15
	21-30	41	20.5
	31-40	43	21.5
	41-50	31	15.5
	51-60	21	10.5
	> 60	34	17
Total		200	100

**Table 2:** Hematological profile of patients

**Parameter**	**Range**	**Number (n)**	**Percentage (%)**
Hemoglobin (g/dL)	< 4.9	107	53.5
	5 - 7.9	84	42
	8 - 10.9	9	4.5
	> 11$	0	0
Total Leukocyte Count	501 - 1000	17	8.5
(/cu mm)	1001 - 2000	50	25
	2001 - 3000	68	34
	3001 - 4000	65	32.5
Platelet Count	< 20,000	51	25.5
(/cu mm)	20,001 - 40,000	31	15.5
	40,001 - 80,000	53	26.5
	80,001 - 120,000	51	25.5
	120,001 - 150,000	13	6.5

**Table 3:** Clinical presentation of study participants

**Clinical Features**	**Number of Patients**	**Frequency (%)**
Generalized weakness	120	60
Shortness of breath	65	32.5
Pallor	65	32.5
Knuckle hyperpigmentation	54	27
Giddiness	48	24
Loss of Appetite	39	19.5
Splenomegaly	28	14
Fever	23	11.5
Bipedal edema	19	9.5

**Table 4:** Etiological distribution of pancytopenia

**Etiology**	**Number of Patients**	**Frequency (%)**
Vitamin B12Deficiency	57	28.5
Hypersplenism	24	12
Dimorphic anaemia	21	10.5
B12Deficiency + Folate deficiency	20	10
HIV	16	8
Chronic Kidney Disease (CKD) groups	13	6.5
Sepsis	12	6
Aplastic anaemia	11	5.5
Chronic Liver Disease (CLD) groups	10	5
Hypothyroidism	9	4.5
Other (Malaria, MDS)	5	2.5

**Table 5:** Statistical associations

**Association**	**Variable 1**	**Variable 2**	**^2^/ p-value**	**Significance**
Gender & B12Deficiency	Male/Female	Presence of Deficiency	p = 0.028	Significant
Age & Weakness	Age Groups	General Weakness	p = 0.05	Significant
Age & Pallor	Age Groups	Presence of Pallor	p = 0.75	Not Significant
